# Risk factors for postpartum alanine aminotransferase elevation in women with chronic hepatitis B infection: a systematic review and meta-analysis

**DOI:** 10.3389/fpubh.2026.1956281

**Published:** 2026-10-14

**Authors:** Chengxuan Yao, Xiang Gao, Yunxia Zhu, Xiaohui Zhang, Shuiweizhi Dai, Jinhua Wang, Yu Chen, Huaibin Zou

**Affiliations:** 1 Fourth Department of Liver Disease Center, Beijing YouAn Hospital, Capital Medical University, Beijing, China; 2 Department of Obstetrics and Gynecology, Beijing YouAn Hospital, Capital Medical University, Beijing, China

**Keywords:** ALT elevation, chronic hepatitis B, hepatitis flares, meta–analysis, postpartum

## Abstract

**Background:**

Postpartum alanine aminotransferase (ALT) elevation is common in women with chronic hepatitis B (CHB) infection and may adversely affect maternal recovery and long-term disease outcomes. However, the risk factors associated with postpartum ALT elevation remain inconsistent across studies.

**Methods:**

We conducted a systematic review and meta-analysis in accordance with the PRISMA guidelines; the registration number is CRD420251208005. PubMed, Web of Science, Embase, Cochrane Library, China National Knowledge Infrastructure (CNKI), and WanFang databases were searched from their inception to 1 January 2026.

**Results:**

A total of 17 cohort studies involving 6,876 women with CHB were included. The meta-analysis demonstrated that hepatitis B e-antigen (HBeAg) positivity (odds ratio [OR] = 3.03; 95% CI [1.91–4.81]), higher baseline hepatitis B virus (HBV) DNA levels (OR = 1.39; 95% CI [1.04–1.86]), and elevated antepartum ALT levels were significantly associated with postpartum ALT elevation. A subgroup analysis revealed that antepartum ALT elevation, defined as a categorical variable (ALT >1 ULN), showed a strong association with postpartum ALT elevation (OR = 5.54, 95% CI 3.78–8.12), whereas continuous ALT increase showed a smaller effect size. Increasing maternal age was associated with a reduced risk of postpartum ALT elevation (OR = 0.93, 95% CI 0.87–0.99).

**Conclusion:**

HBeAg positivity, high baseline HBV DNA levels, and elevated antepartum ALT levels are key risk factors for postpartum ALT elevation in women with CHB. Additionally, younger maternal age may confer increased susceptibility.

**Systematic review registration:**

https://www.crd.york.ac.uk/PROSPERO/view/CRD420251208005, Identifier: CRD420251208005.

## 1 Background

Chronic hepatitis B (CHB) infection remains a significant global public health issue (1, 2). Women with CHB face unique challenges during pregnancy and the postpartum period, which continue to be an important area of clinical research. During pregnancy, the body undergoes various physiological changes to support fetal development, and the majority of pregnant women are in a state of “immune tolerance” to the hepatitis B virus (HBV), resulting in relatively stable liver function (3, 4). However, after childbirth, hormone levels decline rapidly, and the immune system begins to respond aggressively to HBV-infected liver cells, which can lead to hepatitis flares (5). Postpartum alanine aminotransferase (ALT) elevation is not merely a transient laboratory anomaly; it reflects a clinically significant hepatic inflammatory event (6). Some patients may experience increased ALT levels or even elevated bilirubin levels, along with the progression of liver fibrosis. These changes reflect enhanced HBV replication and immune-mediated liver cell damage. If not identified promptly, some patients risk developing persistent hepatitis, advancing liver fibrosis, and potentially liver failure (7, 8). As early as 2015, the WHO guidelines for chronic hepatitis B recommended individualized antiviral therapy based on ALT concentrations (9). Therefore, identifying the high-risk factors for postpartum ALT elevation helps determine the frequency of postpartum monitoring and the timely initiation or resumption of antiviral therapy.

Currently, the clinical understanding of postpartum hepatitis in mothers with CHB is still insufficient. Previous studies have suggested that the development of postpartum hepatitis may be related to various factors, including liver function status during pregnancy and hepatitis B virus (HBV) DNA levels (1, 10, 11). However, these studies vary in sample sizes and have discrepancies in identifying the risk factors, leading to a lack of consensus. Identifying the core risk factors for postpartum hepatitis in mothers with CHB is crucial for accurately identifying high-risk populations, formulating individualized pre-pregnancy assessment plans, and developing postpartum follow-up monitoring strategies. By implementing early interventions in high-risk groups, the incidence of postpartum hepatitis can be effectively reduced, disease progression can be delayed, and maternal–infant outcomes can be improved.

Therefore, this study aims to systematically explore the risk factors associated with postpartum hepatitis in mothers with CHB. The goal is to identify key indicators closely related to postpartum ALT elevation, which serves as a reliable marker for postpartum hepatitis, and to provide evidence-based guidance for optimizing the comprehensive management strategies for mothers with CHB.

## 2 Methods

### 2.1 Study registration

This study was registered with the International Prospective Register of Systematic Reviews (PROSPERO) under the registration number CRD420251208005 and was completed following the Preferred Reporting Items for Systematic Reviews and Meta-Analyses (PRISMA) guidelines.

### 2.2 Search strategy

We searched PubMed, Web of Science, Embase, Cochrane Library, China National Knowledge Infrastructure (CNKI), and WanFang from database inception to 1 January 2026. The Medical Subject Headings (MeSH) terms and keywords used in the search were “Hepatitis B,” “Hepatitis B Virus Infection,” “Hepatitis B Virus,” “HBV,” “Hepatitis,” “Postpartum Period,” “Period, Postpartum,” “Postpartum,” “Postpartum Women,” “Women, Postpartum,” “Puerperium,” “Alanine Transaminase,” “Alanine Aminotransferase,” “ALT,” “ALT Elevation,” “Symptom Flare Up,” “Abnormal Liver Function,” “Hepatitis activity,” and “Hepatitis flare*.” The search strategy is listed in Supplementary Material 1. This study had no language restrictions but excluded review articles and dissertations.

### 2.3 Inclusion and exclusion criteria

The inclusion criteria were as follows: (i) pregnant women with positive HBsAg and/or HBV DNA status for ≥ 6 months; (ii) retrospective or prospective studies excluding case reports, cohort studies with fewer than 10 patients, and postpartum follow-up of less than 1 month. The exclusion criteria were as follows: (i) coinfection with HIV-1, HAV, HCV, HDV, HEV, or sexually transmitted diseases; (ii) associated liver cancer, cirrhosis, or hepatic decompensation; (iii) patients who received nephrotoxic drugs, steroids, cytotoxic drugs, or immunomodulators and underwent liver transplantation, and/or received hemodialysis in the same admission; (iv) lack of sufficient clinical data or incomplete follow-up data; and (v) reviews, dissertations, case reports, and conference abstracts. Two authors (YCX and GX) independently reviewed the titles and abstracts, followed by a full-text screening of eligible studies. Any disagreements were resolved through unified consultation, and the consensus on the study selection was evaluated using Cohen’s kappa.

### 2.4 Data extraction and quality assessment

We extracted relevant information from the literature that met the inclusion and exclusion criteria, including the first author, country and region, cohort data source, study type, sample size, age, definition of ALT elevation, number of patients with ALT elevation, follow-up duration, and risk factors for ALT elevation. Two researchers (YCX and GX) used the Newcastle–Ottawa Scale (NOS) to evaluate the quality of observational cohort studies, with a minimum score of 0 and a maximum score of 9. Scores of more than 7 points were high-quality studies, scores of 5–6 points were medium-quality studies, and scores of 4 points and below were low-quality studies. At the same time, we used risk-of-bias assessment to summarize the risk of each study.

### 2.5 Outcome measurements

The primary outcome was defined as an ALT level exceeding 1 time the upper limit of normal (ULN) at any point after delivery (12). We investigated the potential risk factors for postpartum ALT elevation in women with CHB, including age, baseline HBV DNA, HBeAg positivity, antepartum ALT levels, whether antiviral therapy was administered during pregnancy, and whether antiviral therapy was continued after delivery.

### 2.6 Statistical analysis

We used the “meta” package of R statistical software version 4.0.0 (R Foundation for Statistical Computing, Vienna, Austria) for this meta-analysis. Considering the clinical heterogeneity in the data and the different sample sizes included in the study, the random-effects model was used to pool the quantitative data (13). Heterogeneity was assessed using I^2^ statistics, with 0 to 30% as mild heterogeneity, 30 to 60% as moderate heterogeneity, and 60 to 100% as high heterogeneity. Common risk factors for postpartum liver dysfunction were divided into continuous variables and categorical variables for comparison. Each risk factor included in the meta-analysis was subjected to sensitivity analysis using the leave-one-out method to evaluate the robustness of the results. Funnel plots and Egger’s test were performed for risk factors supported by more than five studies. A *p*-value less than 0.05 was considered statistically significant.

## 3 Results

Initially, we retrieved 1,221 articles, and after excluding duplicate records, there were 865 articles. Based on the title and abstract screening, 833 articles were excluded, and 32 articles were screened through full-text review (Figure 1). Finally, 17 studies were included in this meta-analysis (14–30). The consistency between the two evaluators was very high, with the κ statistic of 0.91.

**Figure 1:**
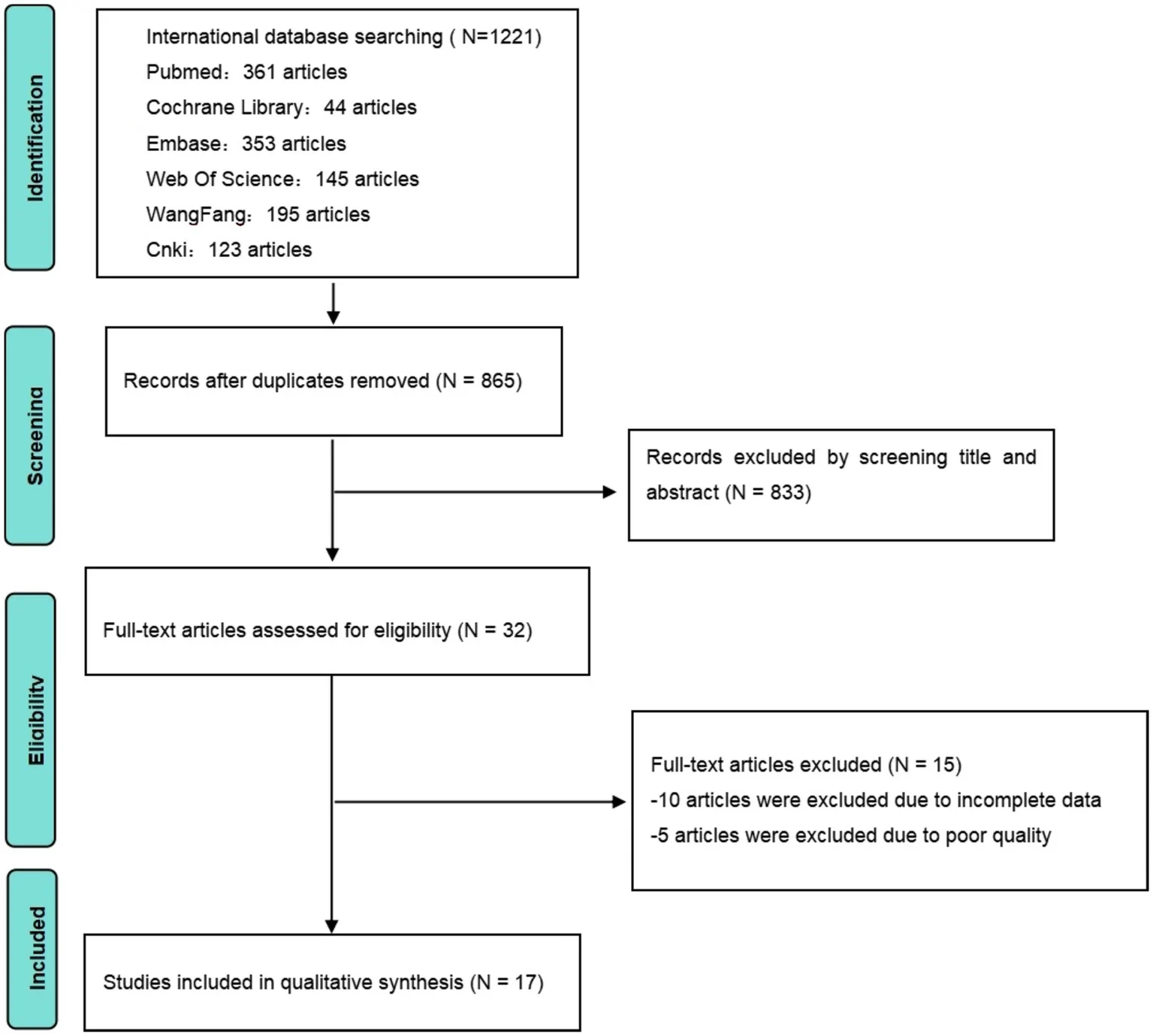
Selection process of the included studies.

### 3.1 Characteristics of the included studies

The characteristics of the 17 included studies are shown in Supplementary Material 2. A total of 16 studies were from China (9 in Beijing, 1 in Zhejiang, 2 in Guangdong, 1 in Sichuan, 1 in Shanghai, 1 in Shaanxi, and 1 in Guangxi), while one study was from Australia. There were 12 prospective cohort studies and 5 retrospective cohort studies. The sample size ranged from 86 to 2,643 patients. The mean age of the included patients ranged from 27.16 to 31.45 years, and the duration of postpartum follow-up ranged from 6 to 48 weeks. Each study included at least one risk factor. According to the NOS tool, the quality of each cohort study was evaluated, which included 4 medium-quality and 13 high-quality studies (Supplementary Material 3). Here, we performed a meta-analysis of risk factors that were evaluated in at least three studies.

### 3.2 Risk factors

#### 3.2.1 Antepartum ALT

Four studies reported the effect of antepartum ALT levels on postpartum ALT elevation in pregnant women with HBV infection. The pooled results showed that an increase in antepartum ALT levels (random-effects model, odds ratio [OR] 1.31; 95% CI [1.12–1.53]) was associated with postpartum ALT elevation (I^2^ = 95.9%; P_heterogeneity_ < 0.0001). For further discussion, a subgroup analysis was performed based on different measurement scales of ALT. The results showed that ALT was used as a categorical variable in two studies (random-effects model, OR 5.54; 95% CI [3.78–8.12]), and there was no heterogeneity between these studies (I^2^ = 0.0%; P_heterogeneity_ = 0.7182). The association was consistent across the two included studies, although the limited number of studies warrants cautious interpretation. Two studies used ALT as a continuous variable (random-effects model, OR 1.05; 95% CI [1.03–1.07]), and the heterogeneity was reduced to 0 (I^2^ = 0.0%; P_heterogeneity_ = 0.9255) (Figure 2). The test for subgroup interaction showed a statistically significant difference between the two analytical approaches (P_interaction_ = 0.000957). The odds ratio (OR) for continuous versus categorical ALT was 0.190 (95% CI, 0.152–0.237), indicating that the pooled effect estimate was substantially lower when ALT was analyzed as a continuous rather than a categorical variable. These findings suggest that the analytical specification of antepartum ALT may influence the magnitude of the estimated association with postpartum ALT elevation.

**Figure 2:**
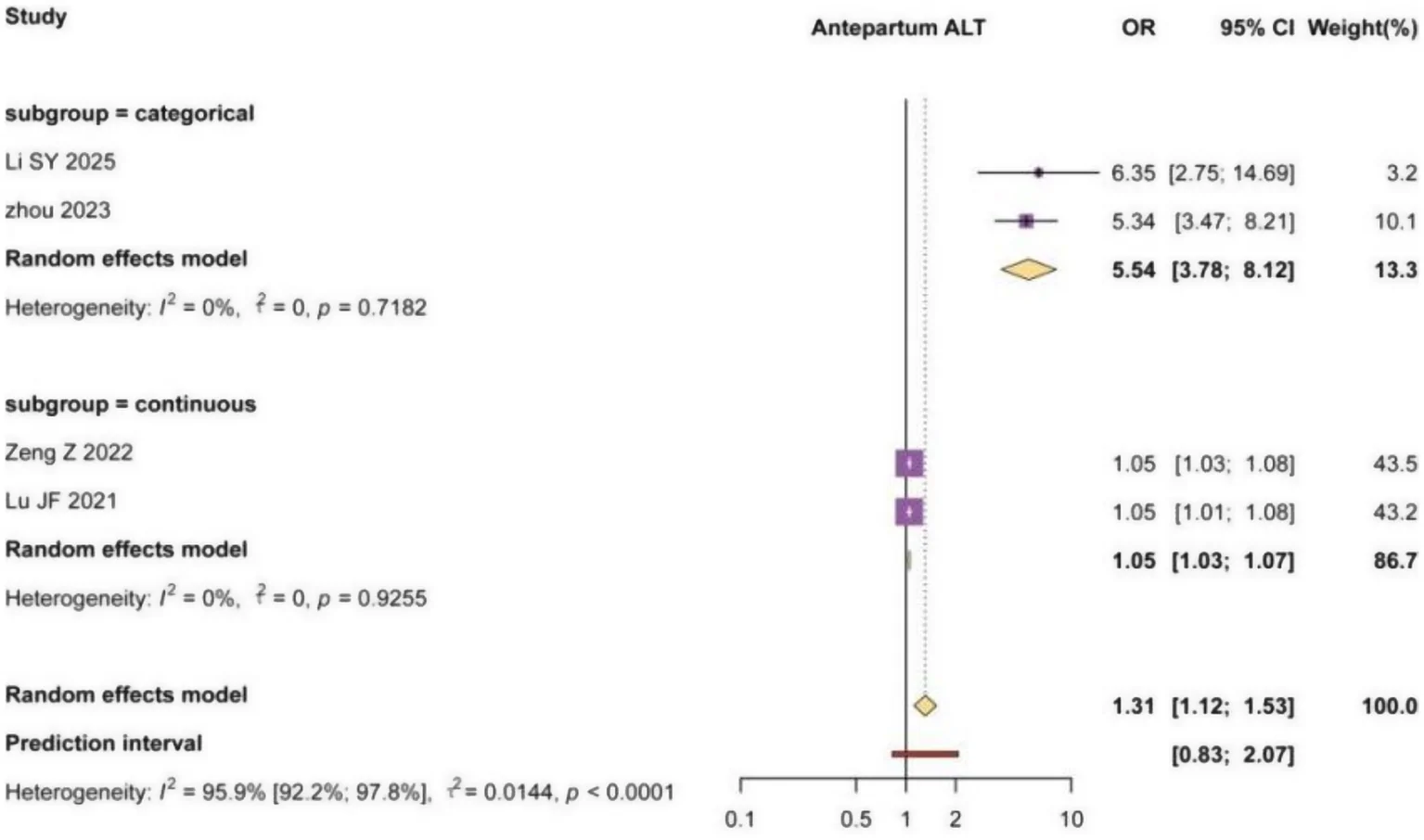
Forest plot of ALT at delivery associated with postpartum ALT elevation.

#### 3.2.2 HBeAg positivity

Five studies explored the effect of HBeAg positivity on postpartum ALT elevation. The pooled results showed that when HBeAg was positive, the risk of postpartum ALT elevation was significantly increased (random-effects model, OR 3.03; 95% CI [1.91–4.81]), with low heterogeneity among studies (I^2^ = 0.0%; P_heterogeneity_ = 0.8800) (Figure 3a). The risk factor was evaluated for publication bias, and the funnel plot was relatively symmetrical; the evidence showed no small-study effect (P_Egger_ = 0.5887) (Figure 1 of Supplementary Material 4).

**Figure 3:**
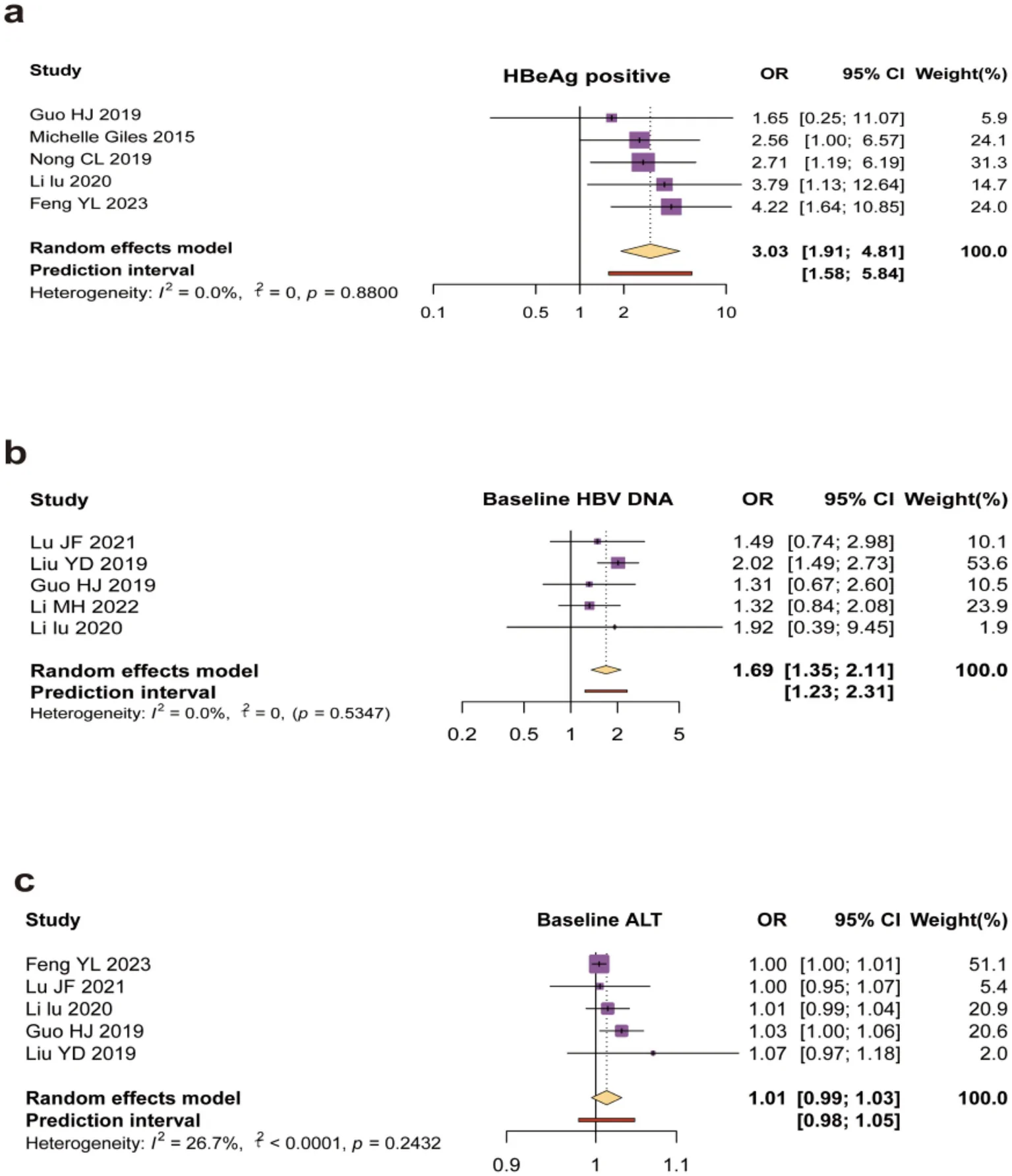
Forest plot and funnel plot of risk factors for postpartum ALT elevation. **(a)** HBeAg positivity; **(b)** baseline HBV DNA; **(c)** baseline ALT.

#### 3.2.3 Baseline HBV DNA

Six studies reported the effect of baseline HBV DNA on postpartum ALT elevation. The pooled results showed that increased baseline HBV DNA (random-effects model, OR 1.39; 95% CI [1.04–1.86]) was associated with postpartum ALT elevation (I^2^ = 66.8%; P_heterogeneity_ = 0.0010) (Figure 2 of Supplementary Material 4). Further sensitivity analysis showed that the OR was between 0.94 and 1.25, indicating that the sensitivity was low. Moreover, after the removal of the study by Nong et al., the heterogeneity of the remaining studies was reduced to 0% (random-effects model, OR 1.64; 95% CI [1.25–2.14]; P_heterogeneity_ = 0.5347) (Figure 3b). Figure 3 of Supplementary Material 4 shows the leave-one-out diagram. Egger’s test showed that the asymmetry of the funnel plot did not reach a statistically significant level (*p* = 0.1059). Trim-and-fill analysis estimated that one study may be missing. The pooled effect size of baseline HBV DNA before and after adjustment was OR = 1.64 (95% CI: 1.25–2.14) and OR = 1.71(95% CI: 1.35–2.15), respectively. The pooled effect size and its statistical significance did not change substantially after the adjustment, suggesting that potential publication bias had little effect on the results of the meta-analysis (Figure 4 of Supplementary Material 4).

#### 3.2.4 Baseline ALT

The combined results of the five studies showed that there was no statistically significant association between the continuous increase in baseline ALT and the postpartum ALT elevation (random-effects model, OR 1.01; 95% CI [0.99–1.03]), and the heterogeneity between studies was low, indicating that there was no obvious inconsistency (I^2^ = 26.7%; P_heterogeneity_ = 0.2432) (Figure 3c). The pooled analysis of five studies showed no statistically significant association between increased baseline ALT as a continuous variable and postpartum ALT elevation (random-effects model, OR = 1.01; 95% CI, 0.99–1.03). Egger’s regression test did not indicate significant funnel plot asymmetry (*p* = 0.1466), suggesting no clear evidence of publication bias. The trim-and-fill method estimated that one study was potentially missing. After adjustment, the pooled OR was 1.006 (95% CI, 0.99–1.02), which was similar to the original estimate, indicating that potential publication bias was unlikely to materially influence the results (Figure 5 of Supplementary Material 4).

#### 3.2.5 Age

Only four studies reported the effect of age. The results showed that there was an inverse association between age and postpartum ALT elevation in HBV patients (random-effects model, OR 0.93; 95% CI [0.87–0.99]), and the results were highly consistent among studies (I^2^ = 0.0%; P_heterogeneity_ = 0.5952) (Figure 4a).

**Figure 4:**
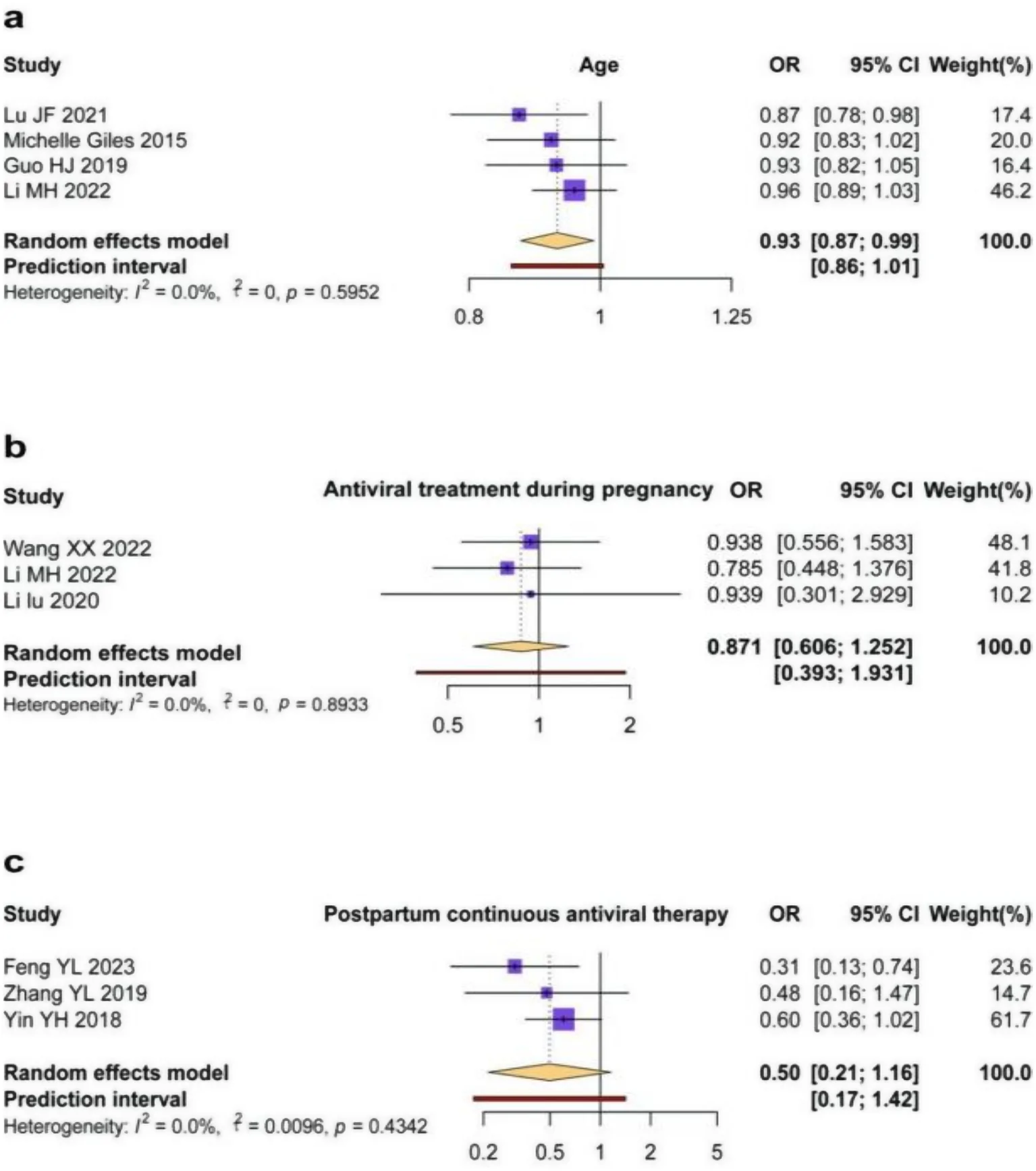
Forest plot of risk factors for postpartum ALT elevation in CHB parturients. **(a)** age; **(b)** antiviral therapy during pregnancy; **(c)** continued postpartum antiviral therapy.

#### 3.2.6 Antiviral treatment during pregnancy

Four studies reported the impact of antiviral therapy during pregnancy, and the results showed that untreated patients during pregnancy had a higher risk of postpartum ALT elevation (random-effects model, OR 0.697; 95% CI [0.475–1.022]; I^2^ = 46.5%; P_heterogeneity_ = 0.1498) (Figure 6 of Supplementary Material 4). We conducted a sensitivity analysis and found that after excluding the study of Quan et al., the heterogeneity decreased to 0% (random-effects model, OR 0.871; 95% CI [0. 606–1.252]; I^2^ = 0.0%; P_heterogeneity_ = 0.8933) (Figure 4b). Figure 7 of Supplementary Material 4 shows the leave-one-out diagram.

#### 3.2.7 Postpartum antiviral therapy

Three studies discussed the impact of continuation of postpartum anti-HBV medication on postpartum ALT elevation. The combined results showed that continued postpartum antiviral therapy was associated with postpartum ALT elevation (random-effects model, OR 0.50; 95% CI [0. 21–1.16]), and the heterogeneity of these three studies was 0% (P_heterogeneity_ = 0.5952) (Figure 4c).

## 4 Discussion

This systematic review and meta-analysis comprehensively evaluated a variety of high-risk factors affecting postpartum ALT elevation in women with CHB. Our results showed that HBeAg positivity, high baseline HBV DNA levels, and elevated antepartum ALT levels were significantly associated with postpartum ALT elevation. We also found that younger mothers had a higher risk of ALT elevation.

This study provides deeper insights into the subgroup analyses of antepartum ALT levels. When elevated antepartum ALT was analyzed as a categorical variable, defined as an ALT level above the upper limit of normal, it showed a strong association with postpartum ALT elevation (OR = 5.54, 95% CI: 3.78–8.12). However, when ALT was analyzed as a continuous variable, an association with postpartum ALT elevation was observed in some studies, although the effect size was considerably smaller (OR = 1.05, 95% CI: 1.03–1.07) compared to the categorical analysis. These findings suggest the possibility of a threshold effect, whereby the risk may increase sharply once ALT exceeds a certain threshold. However, this hypothesis requires validation in larger cohort studies because supporting evidence is currently limited. Moreover, as each subgroup included only two studies, these findings should be regarded as supportive and exploratory evidence rather than being interpreted as definitive or fully robust conclusions. In contrast, the effect of baseline ALT levels on postpartum ALT elevation appeared to be relatively small (OR = 1.01, 95% CI: 0.99–1.03), which may be attributable to initial differences between patients who received regular treatment during pregnancy.

This study also found that HBeAg positivity was significantly associated with postpartum ALT elevation, with a pooled OR of 3.03 (95% CI: 1.91–4.81), and no statistically significant heterogeneity was observed among the included studies. HBeAg positivity generally indicates active viral replication or a relatively higher viral burden. Changes in immune status during pregnancy and the postpartum period may jointly influence HBV-related hepatic inflammation. Therefore, HBeAg-positive pregnant women may represent a high-risk group warranting closer postpartum monitoring of liver function. An elevated baseline HBV DNA level was likewise associated with postpartum ALT elevation, with a pooled OR of 1.39 (95% CI: 1.04–1.86). This finding suggests that a higher level of viral replication may serve as an important risk marker for postpartum hepatic dysfunction. Notably, the timing of baseline HBV DNA measurement was not entirely consistent across the six relevant studies included in this analysis. In the study by Nong et al., antiviral therapy was initiated at 24 weeks of gestation, whereas treatment was generally initiated at 28 weeks of gestation or later in the other five studies. Differences in the timing of treatment initiation may have affected baseline viral load, virological changes during pregnancy, and the subsequent observation of postpartum ALT elevation, thereby contributing to clinical heterogeneity across studies. Following sensitivity analysis, the heterogeneity decreased to zero, suggesting that certain studies may have contributed substantially to between-study heterogeneity.

A noteworthy finding was that older age appeared to be associated with a lower risk of postpartum ALT elevation (OR = 0.93, 95% CI: 0.87–0.99), suggesting that the risk of postpartum ALT elevation may decrease with increasing maternal age. Younger women may have more robust immune responses and, consequently, may experience a more pronounced postpartum immune rebound. In contrast, older pregnant women may have had a longer duration of HBV infection, with some potentially having entered the immune-tolerant phase or inactive phase, during which the host immune response to viral replication is relatively less active. Additionally, differences between younger and older pregnant women in the frequency of antenatal care, liver function monitoring, hepatoprotective medication use, decisions regarding antiviral therapy, and postpartum follow-up may also influence the observed association between age and postpartum ALT elevation. Given the limited evidence available, this finding should be interpreted as exploratory. Nevertheless, it highlights the potential importance of closer postpartum monitoring of liver function in younger pregnant women.

Regarding antiviral therapy during pregnancy, the pooled analysis showed a trend toward a lower likelihood of postpartum ALT elevation in the antiviral treatment group compared with the control group, with a pooled OR of 0.697 (95% CI: 0.475–1.022); however, this difference did not reach statistical significance.

For continuation of antiviral therapy after delivery, the pooled results indicated that continued postpartum antiviral treatment was associated with a lower likelihood of postpartum ALT elevation, with a pooled OR of 0.50 (95% CI: 0.21–1.16). Although the effect estimate suggested a potentially protective trend, the confidence interval was relatively wide and crossed the null value of 1. Therefore, a statistically significant protective effect cannot yet be established. Despite the limited strength of the available evidence, this potential protective trend warrants further investigation.

This meta-analysis has several limitations. First, among the 17 included cohorts, only one enrolled participants who were not of Chinese nationality, and the majority of studies were conducted in China. Therefore, the findings primarily reflect the clinical characteristics and management patterns of pregnant women with chronic hepatitis B in China. Caution is warranted when generalizing these results to postpartum women with chronic hepatitis B from non-Chinese populations. Second, differences in the postpartum follow-up windows and outcome definitions across studies represent important potential sources of clinical heterogeneity. In addition, several unmeasured confounding factors, including concomitant medications, pregnancy-related comorbidities, and the degree of hepatic inflammatory activity during pregnancy, were not consistently reported or clearly accounted for in the individual studies and may have influenced the pooled estimates. Finally, the number of studies included in the pooled analysis of each factor was limited. Although risk-of-bias assessments were performed, they cannot fundamentally eliminate the potential influence of residual bias and confounding. Accordingly, these findings should be regarded as exploratory and hypothesis-generating. Further large-scale, multicenter studies with standardized variable definitions and adequate adjustment for confounders are needed to validate the identified risk factors and to clarify the true relationship between antiviral therapy and postpartum ALT elevation. In particular, meta-analyses of individual patient data may provide more robust evidence on this topic.

## Data Availability

The raw data supporting the conclusions of this article will be made available by the authors, without undue reservation.
