# Supplementary material for: Risk factors for postpartum alanine aminotransferase elevation in women with chronic hepatitis B infection: a systematic review and meta-analysis
Source: Front Public Health. 2026 Oct 14;14:1956281. doi: 10.3389/fpubh.2026.1956281 (PMC13713853; doi:10.3389/fpubh.2026.1956281)
Supplement: Supplementary file 1 [file Data_Sheet_1.PDF]

Supplementary Material 1. Search strategy

Detailed search strategy in Pubmed

#1 "Hepatitis B"[MeSH Terms] AND "Postpartum Period"[MeSH Terms]

#2 ("hepatitis b"[Title/Abstract] OR "Hepatitis B Virus Infection"[Title/Abstract] OR "Hepatitis B Virus"[Title/Abstract] OR "HBV"[Title/Abstract]) AND ("postpartum period"[Title/Abstract] OR "period postpartum"[Title/Abstract] OR "Postpartum"[Title/Abstract] OR "Postpartum Women"[Title/Abstract] OR "women postpartum"[Title/Abstract] OR "Puerperium"[Title/Abstract])

#3 ("Hepatitis B"[MeSH Terms] AND "Postpartum Period"[MeSH Terms]) OR (("Hepatitis B"[Title/Abstract] OR "Hepatitis B Virus Infection"[Title/Abstract] OR "Hepatitis B Virus"[Title/Abstract] OR "HBV"[Title/Abstract]) AND ("Postpartum Period"[Title/Abstract] OR "period postpartum"[Title/Abstract] OR "Postpartum"[Title/Abstract] OR "Postpartum Women"[Title/Abstract] OR "women postpartum"[Title/Abstract] OR "Puerperium"[Title/Abstract]))

#4 "Hepatitis B"[MeSH Terms] OR "Hepatitis B"[Title/Abstract] OR "Hepatitis B Virus Infection"[Title/Abstract] OR "Hepatitis B Virus"[Title/Abstract] OR "HBV"[Title/Abstract]

#5 "Postpartum Period"[MeSH Terms] OR "Postpartum Period"[Title/Abstract] OR "period postpartum"[Title/Abstract] OR "Postpartum"[Title/Abstract] OR "Postpartum Women"[Title/Abstract] OR "women postpartum"[Title/Abstract] OR "Puerperium"[Title/Abstract]

#6 ("Hepatitis B"[MeSH Terms] OR ("Hepatitis B"[Title/Abstract] OR "Hepatitis B

Virus Infection"[Title/Abstract] OR "Hepatitis B Virus"[Title/Abstract] OR "HBV"[Title/Abstract])) AND ("Postpartum Period"[MeSH Terms] OR ("Postpartum Period"[Title/Abstract] OR "period postpartum"[Title/Abstract] OR "Postpartum"[Title/Abstract] OR "Postpartum Women"[Title/Abstract] OR "women postpartum"[Title/Abstract] OR "Puerperium"[Title/Abstract]))

Detailed search strategy in Cochrane Library

#1 MeSH descriptor: [Hepatitis B] explode all trees

#2 (Hepatitis B):ti,ab,kw OR (Hepatitis B Virus Infection):ti,ab,kw OR (Hepatitis B Virus):ti,ab,kw OR (HBV):ti,ab,kw

#3 MeSH descriptor: [Postpartum Period] explode all trees

#4 (Postpartum Period):ti,ab,kw OR (Period, Postpartum):ti,ab,kw OR (Postpartum):ti,ab,kw OR (Postpartum Women):ti,ab,kw OR (Women, Postpartum):ti,ab,kw

#5 (Women, Postpartum):ti,ab,kw AND (Puerperium):ti,ab,kw

#6 #1 OR #2

#7 #3 OR #4 OR #5

#8 #6 AND #7

#9 MeSH descriptor: [Alanine Transaminase] explode all trees

#10 (Alanine Transaminase):ti,ab,kw OR (Alanine Aminotransferase):ti,ab,kw OR (Alanine 2 Oxoglutarate Aminotransferase):ti,ab,kw OR (Glutamic-Alanine Transaminase):ti,ab,kw OR (Glutamic-Pyruvic Transaminase):ti,ab,kw

#11 (Glutamic-Pyruvic Transaminase):ti,ab,kw OR (SGPT):ti,ab,kw OR (ALT):ti,ab,kw OR (ALT Elevation):ti,ab,kw OR (Symptom Flare Up):ti,ab,kw

#12 (Symptom Flare Up):ti,ab,kw OR (Abnormal Liver Function):ti,ab,kw OR (Hepatitis activ*):ti,ab,kw OR (Hepatitis flare*):ti,ab,kw OR (Transaminase, Alanine):ti,ab,kw

#13 (Transaminase, Alanine):ti,ab,kw OR (Aminotransferase, Alanine):ti,ab,kw OR (Glutamic Alanine Transaminase):ti,ab,kw OR (Transaminase, Glutamic-Alanine):ti,ab,kw OR (Glutamic Pyruvic Transaminase):ti,ab,kw

#14 (Glutamic Pyruvic Transaminase):ti,ab,kw OR (Transaminase, Glutamic-Pyruvic):ti,ab,kw

#15 #9 OR #10 OR #11 OR #12 OR #13 OR #14

#16 #15 AND #8

Detailed search strategy in Embase

#1 hepatitis b'/exp OR 'hepatitis b' OR 'hepatitis b virus infection'/exp OR 'hepatitis b virus infection' OR 'hepatitis b virus'/exp OR 'hepatitis b virus' OR 'hbv'/exp OR 'hbv'

#2 'postpartum period' OR 'period, postpartum' OR 'postpartum' OR 'postpartum women' OR 'women, postpartum' OR 'puerperium'

#3 'alanine transaminase' OR 'alanine aminotransferase' OR 'glutamic-alanine transaminase' OR 'glutamic-pyruvic transaminase' OR 'sgpt' OR 'alt' OR 'alt elevation' OR 'symptom flare up' OR 'abnormal liver function' OR 'hepatitis activ*' OR 'hepatitis flare*' OR 'transaminase, alanine' OR 'aminotransferase, alanine' OR

'glutamic?alanine transaminase' OR 'transaminase, glutamic-alanine' OR 'glutamic pyruvic transaminase' OR 'transaminase, glutamic-pyruvic' OR 'alanine-2-oxoglutarate aminotransferase' OR 'alanine 2 oxoglutarate aminotransferase' OR 'aminotransferase, alanine-2-oxoglutarate'

#4 #1 AND #2 AND #3

Detailed search strategy in Web of science

#1 " Hepatitis B" OR " Hepatitis B Virus Infection" OR " Hepatitis B Virus" OR " HBV" (主题)

#2 " Postpartum Period" OR “ Period, Postpartum” OR “ Postpartum” OR “ Postpartum Women” OR “ Women, Postpartum” OR “ Puerperium” (主题)

#3 Alanine Transaminase OR “ Alanine Aminotransferase” OR “ Alanine-2-Oxoglutarate Aminotransferase” OR “ Glutamic-Alanine Transaminase” OR “ Glutamic-Pyruvic Transaminase” OR “ SGPT” OR “ ALT” OR “ ALT Elevation” OR “ Symptom Flare Up” OR “ Abnormal Liver Function” OR “ Hepatitis activ*” OR “ Hepatitis flare*” OR “ Transaminase, Alanine” OR “ Aminotransferase, Alanine” OR “ Glutamic Alanine Transaminase” OR “ Transaminase, Glutamic-Alanine” OR “ Glutamic Pyruvic Transaminase” OR “ Transaminase, Glutamic-Pyruvic” OR “ Alanine-2-Oxoglutarate Aminotransferase” OR “ Alanine 2 Oxoglutarate Aminotransferase” OR “ Aminotransferase, Alanine-2-Oxoglutarate” (主题)

#4 #3 AND #2 AND #1

Detailed search strategy in CNKI

#1（主题：乙型肝炎）（OR（篇关摘：乙型肝炎＋乙肝＋乙型肝炎病毒＋乙型肝炎患者+‘乙型肝炎病毒（hbV）’+乙型肝炎病毒感染+‘乙型肝炎（乙肝）’（精确））

#2（主题：肝功能异常）（OR（篇关摘：丙氨酸转氨酶+‘丙氨酸转氨酶（alt）’+丙氨酸转氨酶升高+丙氨酸转氨酶异常+谷氨酸丙氨酸转氨酶+丙氨酸-2-氧戊二酸盐转氨酶+丙氨酸氨基转移酶+谷氨酸-丙氨酸转氨酶＋SGPT＋症状爆发+肝功能异常（精确)）

#3（主题：产后期）（OR（篇关摘：产后期+产后期疾病+围产后期+产褥期+产后妇女+产后（精确））

#4 #1 AND #2 AND #3

Detailed search strategy in Wangfang

#1 主题:(乙型肝炎 OR 乙肝 OR 乙型肝炎病毒 OR 乙型肝炎患者 OR hbv)

#2 主题:(产后期 OR 产褥期 OR 产后妇女 OR 产后 OR 围产后期)

#3 主题:(丙氨酸转氨酶 \+ 谷氨酸丙氨酸转氨酶 \+ 丙氨酸\-2\-氧戊二酸盐转氨酶 \+ 丙氨酸氨基转移酶 \+ 谷氨酸\-丙酮酸转氨酶 OR SGPT OR 症状爆发 OR 肝功能异常 OR 肝炎活动)

#4 #1 AND #2 AND #3
