# Supplementary material for: Risk factors for postpartum alanine aminotransferase elevation in women with chronic hepatitis B infection: a systematic review and meta-analysis
Source: Front Public Health. 2026 Oct 14;14:1956281. doi: 10.3389/fpubh.2026.1956281 (PMC13713853; doi:10.3389/fpubh.2026.1956281)
Supplement: Supplementary file 2 [file Data_Sheet_2.PDF]

Supplementary Material 2. Baseline characteristics and risk factors of the 17 cohort studies

First author	Year	Country	Source of cohort	Study design	Sample size (Abnormal / Total)	Age (years)	Definition of outcome	Postpartum Follow-up (weeks)	Risk factors
Li SY[1]	2025	China Zhejiang	Hangzhou First People's Hospital	Prospec-tive	91/160	NA	ALT level ≥ 1×ULN	48	Antepartum ALT
Zhou MF[2]	2023	China Beijing	Beijing Di tan Hospital, Capital Medical University	Retrospe ctive	257/ 2643	28.0±4.0	ALT level ≥ 5×ULN	28	Antepartum ALT
Feng YL[3]	2023	China Shanxi	First Affiliated Hospital of Xi'an Jiaotong University	Retrosp-ective	63/131	29.60 ± 3.63	ALT level ≥ 1×ULN	12	Gestational age (years), Baseline HBV DNA, Baseline HBsAg titer, Baseline HBeAg titer, HBeAg positive, Baseline ALT level, Baseline ALT abnormality, Continuous TDF treatment, Postpartum follow-up duration, A decline of postpartum HBeAg ⩾ 50% from baseline, Postpartum HBeAg seroconversion
Zeng Z[4]	2022	China Beijing	Beijing Di tan Hospital, Capital Medical University	Prospec-tive	231/533	29. 50±3. 7	ALT level ≥ 2×ULN	48	Antepartum ALT, Antepartum ALB, Antepartum HBeAg titer, Antepartum PLT, Antepartum NE%

Wang XX[5]	2022	China Beijing	Beijing You’an Hospital Affiliated to Capital Medical University	Prospec-tive	164/332	28.4±4.20	ALT level ≥ 1×ULN	48	Antiviral therapy during pregnancy
Quan M[6]	2022	China Beijing	Beijing Ditan hospital, Capital Medical University	Prospec-tive	355/614	NA	ALT level ≥ 1×ULN	48	Antiviral therapy during pregnancy
Lu JF[7]	2021	China Beijing	Beijing You’an Hospital	Prospec-tive	56/153	28.80 ± 3.88	ALT level ≥ 2×ULN	6	Age, Genotype, Baseline ALT, HBeAg positive, Baseline HBV DNA, Antepartum ALT, Antepartum HBV DNA, Postpartum HBeAg positive, Postpartum HBV DNA
Li MH[8]	2022	China Beijing	Beijing Di tan Hospital, Capital Medical University	Prospec-tive	67/374	30.74 ± 3.85	ALT level ≥ 2×ULN	24	Age, Gestational weeks, Mode of delivery (self-delivery), Delivery times, HBV DNA level at enrollment, HBV DNA level before delivery, Antiviral therapy, Withdrawal of treatment immediately after delivery
Yu c[9]	2020	China Sichuan	Nantong Third Hospital Affiliated to Nantong University and affiliated hospital of nantong university	Prospec-tive	50/93	27.16 ± 3.83	ALT level ≥ 1×ULN	24	Previous anti-viral infection, coexisting autoimmune diseases, weeks of pregnancy at which antiviral therapy was initiated, tenofovir (TDF) therapy, duration of antiviral therapy, HBeAg titer when antiviral drugs were discontinued

Li L[10]	2020	China Beijing	Beijing YouAn Hospital, Capital Medical University	Retrosp-ective	317	29.3±4.2	ALT level ≥ 1.5×ULN	48	Baseline ALT, Baseline AST, Baseline TBIL, Baseline DBIL, HBeAg positive, Baseline HBV DNA detected, Antiviral therapy during pregnancy, Gestational diabetes mellitus
Wang M[11]	2019	China Beijing	Beijing You'an Hospital Affiliated to Capital Medical University	Prospec-tive	46/109	28.60 ±3.50	ALT level ≥ 1×ULN	48	IL-10
Liu YD[12]	2019	China Shanghai	Shanghai Public Health Clinical Center	Prospec-tive	61/198	30.69 ± 5.85	ALT level ≥ 2×ULN	48	Baseline ALT, Baseline HBcrAg titer, Baseline HBsAg titer, Baseline HBeAg titer, Baseline HBV DNA
Guo HJ[13]	2019	China Guangdon-g	Nanfang Hospital Affiliated to Southern Medical University and Guangzhou Eighth People 's Hospital	Retrosp-ective	30/188	NA	ALT level ≥ 2×ULN	24	Age, Taking TDF, Taking telbivudine, HBeAg positive, Baseline HBV DNA, Baseline ALT, Parity, premature delivery, mode of delivery (Shaping palace production compared to other ways)
Nong CL[14]	2019	China Guangxi	The Fourth Affiliated Hospital of Guangxi Medical University	Retrosp-ective	212/476	29.76 ± 3.84	ALT level ≥ 1×ULN	24	Abnormal ALT during pregnancy, Baseline HBV DNA, HBeAg positive
Zhang	2019	China	Shenzhen Baoan	Prospec-	22/86	NA	ALT level ≥	6	Immediate postpartum withdrawal / 6 weeks

YL[15]		Guangdon-g	District Maternal and Child Health Hospital Obstetrics	tive			1×ULN		postpartum withdrawal
Yin YH[16]	2018	China Beijing	Fifth Medical Center, General Hospital of Chinese PLA	Retrospective	86/343	29.45± 4.80	ALT level ≥ 1×ULN	48	Immediate postpartum withdrawal / 6 weeks postpartum withdrawal
Michelle Giles[17]	2015	Australia	Monash Medical Centre and the Women's Hospital	Prospec-tive	45/126	31.45±0.43	ALT level ≥ 2×ULN	12	Age, ALT, Baseline HBsAg titer, HBeAg positive, Country of birth, Gravida, parity, Baseline HBeAg titer, Year of HBV diagnosis, HBeAb positive, HBV DNA

References

1. Li S, Jiang Y, Shi J, Jiang X, Li Y, Ma Z, et al. Impact of stopping Tenofovir disoproxil fumarate at various times on elevated postpartum ALT levels and breastfeeding in pregnant women with chronic hepatitis B: a prospective observational cohort study. BMC Pregnancy Childbirth. 2025;25:900. https://doi.org/10.1186/s12884-025-08078-x.

2. Zhou M, Cai H, Yi W, Gao X. A Nonlinear Relationship Between ALT Levels at Delivery and the Risk of Postpartum ALT Flares in Pregnant Women with Chronic Hepatitis B. Int J Med Sci. 2023;20:247–53. https://doi.org/10.7150/ijms.79663.

3. Feng Y, Yao N, Shi L, Zhu Y, Liu J, He Y, et al. Efficacy and safety of long-term postpartum antiviral therapy in hepatitis B virus-infected mothers receiving prophylactic tenofovir disoproxil fumarate treatment. European Journal of Gastroenterology & Hepatology. 2023;35:212–8. https://doi.org/10.1097/MEG.0000000000002476.

4. Zeng Z, Zhou MF, Bi XY, Lin YJ, Yang L, Deng W, et al. Real-world study on predictors of postpartum hepatitis in pregnant women with chronic HBV infection. Chinese

Journal of Experimental and Clinical Virology. 2022;36:696–700.

5. Wang X, Song A, Lin X, Lu J, Zheng S, Ma L, et al. Clinical characteristics of hepatitis flares during pregnancy and postpartum in Chinese chronic hepatitis B virus carriers—a prospective cohort study of 417 cases. Front Immunol. 2022;13:1031291. https://doi.org/10.3389/fimmu.2022.1031291.

6. Quan M, Liu X-M, Liu C, Li W, Xing H-C. Antiviral Therapy for Prevention of Perinatal Hepatitis B Virus Transmission Reduces the Incidence of Postpartum Hepatitis Flare. BioMed Research International. 2022;2022:7046955. https://doi.org/10.1155/2022/7046955.

7. Lu J, Wang X, Zhu Y, Ma L, Zheng S, Hu Z, et al. Clinical and Immunological Factors Associated with Postpartum Hepatic Flares in Immune-Tolerant Pregnant Women with Hepatitis B Virus Infection Treated with Telbivudine. Gut and Liver. 2021;15:887–94. https://doi.org/10.5009/gnl21001.

8. Li Z, Xie B, Yi N, Cai H, Yi W, Gao X. Efficacy and safety of tenofovir disoproxil fumarate or telbivudine used throughout pregnancy for the prevention of mother-to-child transmission of hepatitis B virus: A cohort study. European Journal of Obstetrics & Gynecology and Reproductive Biology. 2022;276:102–6. https://doi.org/10.1016/j.ejogrb.2022.07.009.

9. Yu C, Gu YL, Gu GF, Gu EL. Analysis of related factors of hepatitis activity after discontinuation of antiviral therapy in pregnant women with chronic hepatitis B virus carrying status. The Journal of Practical Medicine. 2020;36:3121–5.

10. Li L, Zou H, Xu M, Li S, Zhu Y, Zheng S, et al. Risk factors related to postpartum hepatic inflammation in pregnant women with chronic hepatitis B. J Int Med Res. 2020;48:0300060520966439. https://doi.org/10.1177/0300060520966439.

11. Wang M, Hou Y, Meng S-H, Yang B, Yang P, Zhang H, et al. Abnormal IL-10 levels were related to alanine aminotransferase abnormalities during postpartum in HBeAg positive women with chronic hepatitis B. Medicine. 2019;98:e17969. https://doi.org/10.1097/MD.0000000000017969.

12. Liu YD, Liu M. Construction of a risk prediction model for postpartum hepatitis in pregnant women with chronic HBV infection who received mother-to-child blocking therapy. Progress in Obstetrics and Gynecology. 2024;33:196–200, 204. https://doi.org/10.13283/j.cnki.xdfckjz.2024.03.008.

13. Effect of withdrawal of antiviral therapy during pregnancy on postpartum liver function in pregnant women with chronic hepatitis B virus infection during delivery.

14. Nong CL, Guo Q, Wei QF, Huang JL, Peng XH. Analysis of related influencing factors of postpartum ALT abnormality in pregnant women with chronic hepatitis B virus infection. China Practical Medicine. 2019;14:57–9. https://doi.org/10.14163/j.cnki.11-5547/r.2019.21.031.

15. Zhang YL, Zeng XP. Effect of antiviral drug withdrawal time on postpartum liver function in pregnant women with HBV infection. Journal of North Pharmacy. 2019;16:95–6.

16. Yin YH, Dong ML, Shi M, Wang M. Effect of antiviral withdrawal time on postpartum liver function and mother-to-child transmission in pregnant women with chronic hepatitis B virus infection. Chinese Journal of Infection and Chemotherapy. 2018;18:263–6. https://doi.org/10.16718/j.1009-7708.2018.03.004.

17. Giles M, Visvanathan K, Lewin S, Bowden S, Locarnini S, Spelman T, et al. Clinical and virological predictors of hepatic flares in pregnant women with chronic hepatitis B. Gut. 2015;64:1810–5. https://doi.org/10.1136/gutjnl-2014-308211.
