# Supplementary material for: Risk factors for postpartum alanine aminotransferase elevation in women with chronic hepatitis B infection: a systematic review and meta-analysis
Source: Front Public Health. 2026 Oct 14;14:1956281. doi: 10.3389/fpubh.2026.1956281 (PMC13713853; doi:10.3389/fpubh.2026.1956281)
Supplement: Supplementary file 3 [file Data_Sheet_3.PDF]

First author/year	Selection				Comparability	Outcome			Total scores
	Representa-tiveness of the exposed cohort	Selectio-n of the non-exposed cohort	Ascertai-nment of exposur-e	Demonstration that outcome of interest was not present at start of study	Comparabi-lity of cohorts on the basis of the design or analysis	Assess-ment of outcome	Was follow-up long enough for outcomes to occur	Adequa-cy of follow up of cohorts	
Zhou MF/2023	☆	☆	☆	☆	☆☆	☆	☆	☆	9
Yin YH/2018	☆	☆	☆	-	-	☆	☆	-	5
Wang XX/2022	☆	☆	☆	☆	☆☆	☆	☆	☆	9
Quan M/2022	☆	☆	☆	☆	☆☆	☆	☆	☆	9
Wang M/2019	☆	☆	☆	☆	☆	☆	☆	☆	8
Lu JF/2021	☆	☆	☆	☆	-	☆	-	☆	6
Li SY/2025	☆	☆	☆	☆	☆	☆	☆	☆	8
Michelle Giles/2015	☆	☆	☆	☆	☆☆	☆	☆	☆	9
Zhang YL/2019	☆	☆	☆	-	-	☆	-	☆	5
Yu c/2020	☆	☆	☆	☆	☆	☆	☆	☆	8
Liu YD/2019	☆	☆	☆	☆	☆	☆	☆	☆	8
Zeng Z/2022	☆	☆	☆	☆	☆☆	☆	☆	☆	9
Guo HJ/2019	☆	☆	☆	☆	☆	☆	☆	☆	8
Feng YL/2023	☆	☆	☆	☆	☆	☆	☆	☆	8
Li MH/2022	☆	☆	☆	☆	☆☆	☆	☆	☆	9
Nong CL/2019	☆	☆	☆	☆	-	☆	-	☆	6
Li L/2020	☆	☆	☆	☆	☆	☆	☆	☆	8

Table 1. Newcastle-Ottawa Quality Assessment Scale of the 17 cohort studies.

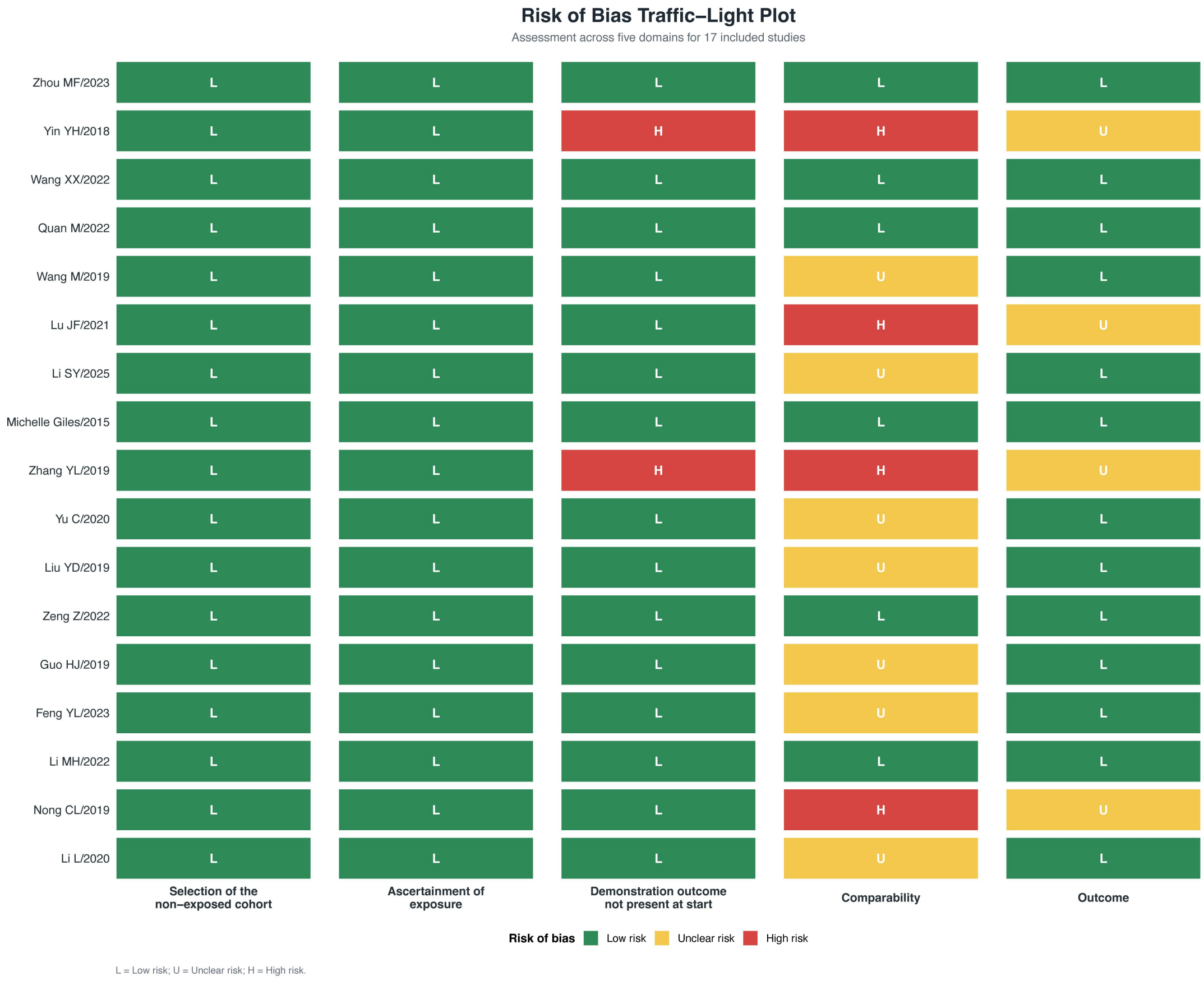

Fig 1. Risk of bias traffic-light plot of 17 studies.
