# Supplementary material for: Risk factors for postpartum alanine aminotransferase elevation in women with chronic hepatitis B infection: a systematic review and meta-analysis
Source: Front Public Health. 2026 Oct 14;14:1956281. doi: 10.3389/fpubh.2026.1956281 (PMC13713853; doi:10.3389/fpubh.2026.1956281)
Supplement: Supplementary file 4 [file Data_Sheet_4.PDF]

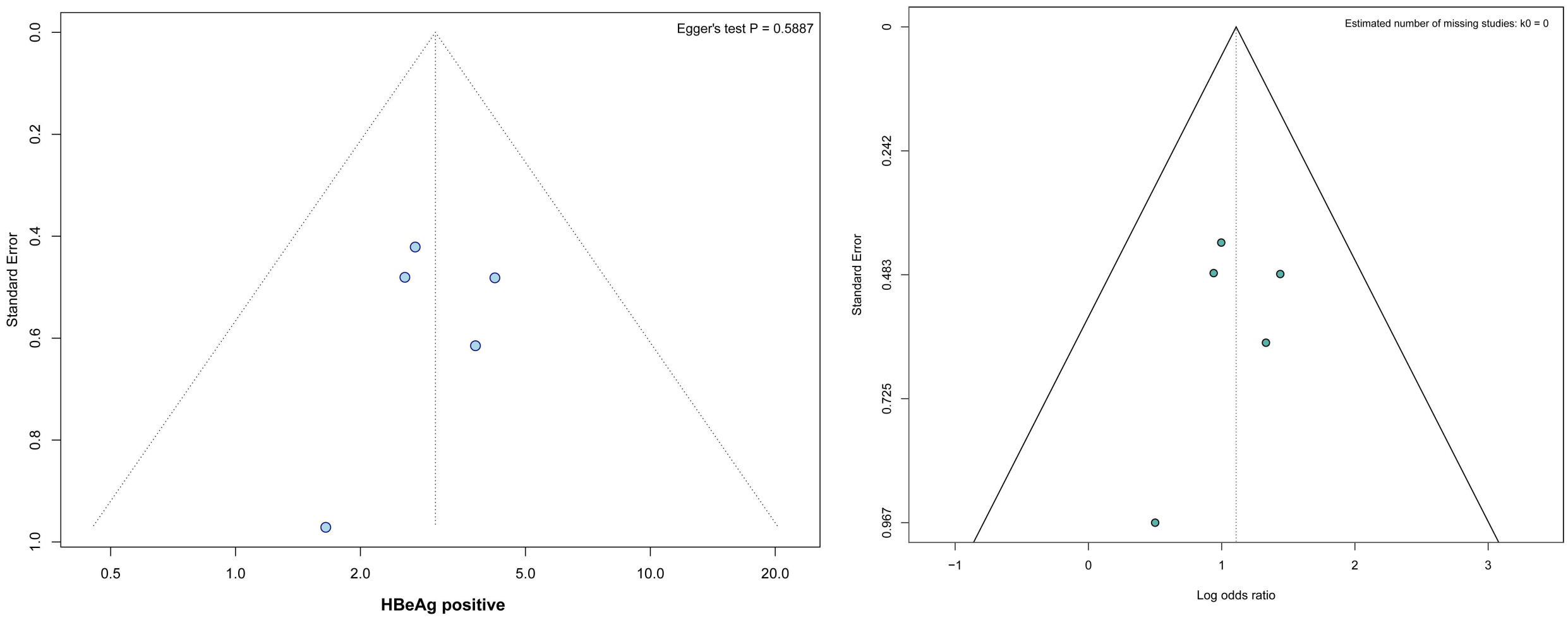

Fig 1. Funnel plot and publication bias assessment using Egger's regression test and the trim-and-fill method for the association between HBeAg positive and postpartum ALT elevation.

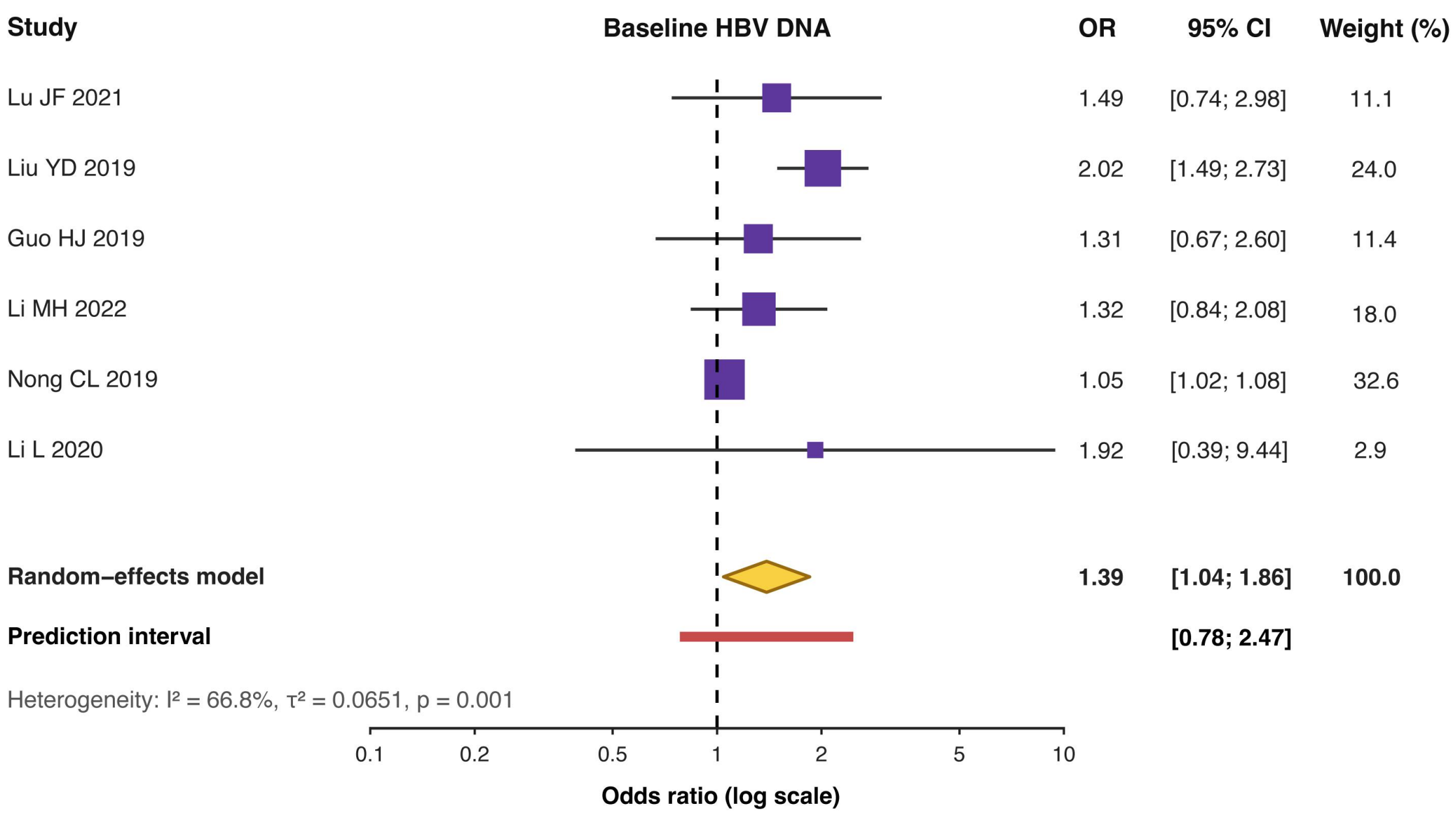

Fig 2. Forest plot and funnel plot of Baseline HBV DNA for postpartum ALT elevation.

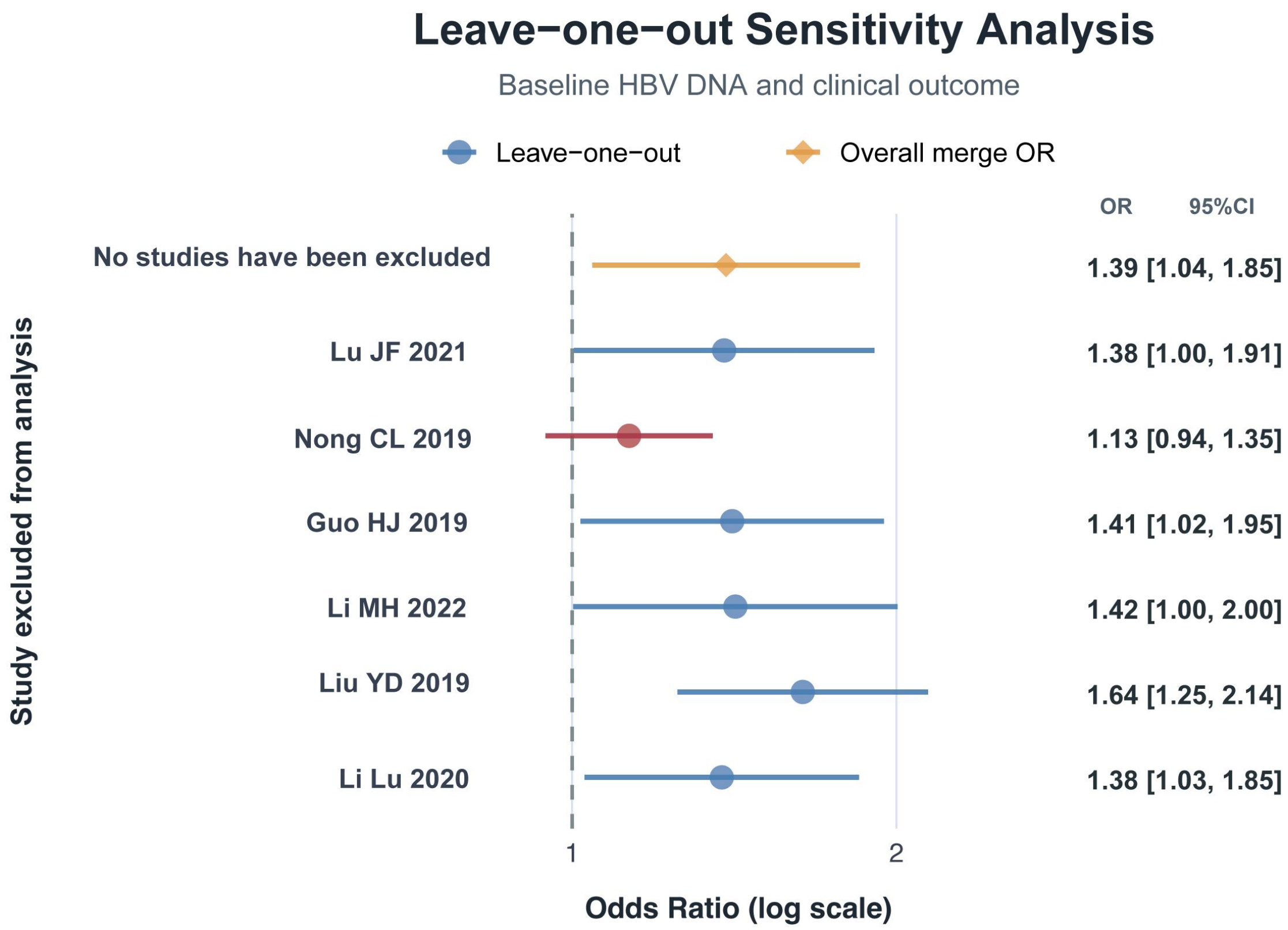

Fig 3. Leave-one-out sensitivity analysis of the meta-analysis of Baseline HBV DNA.

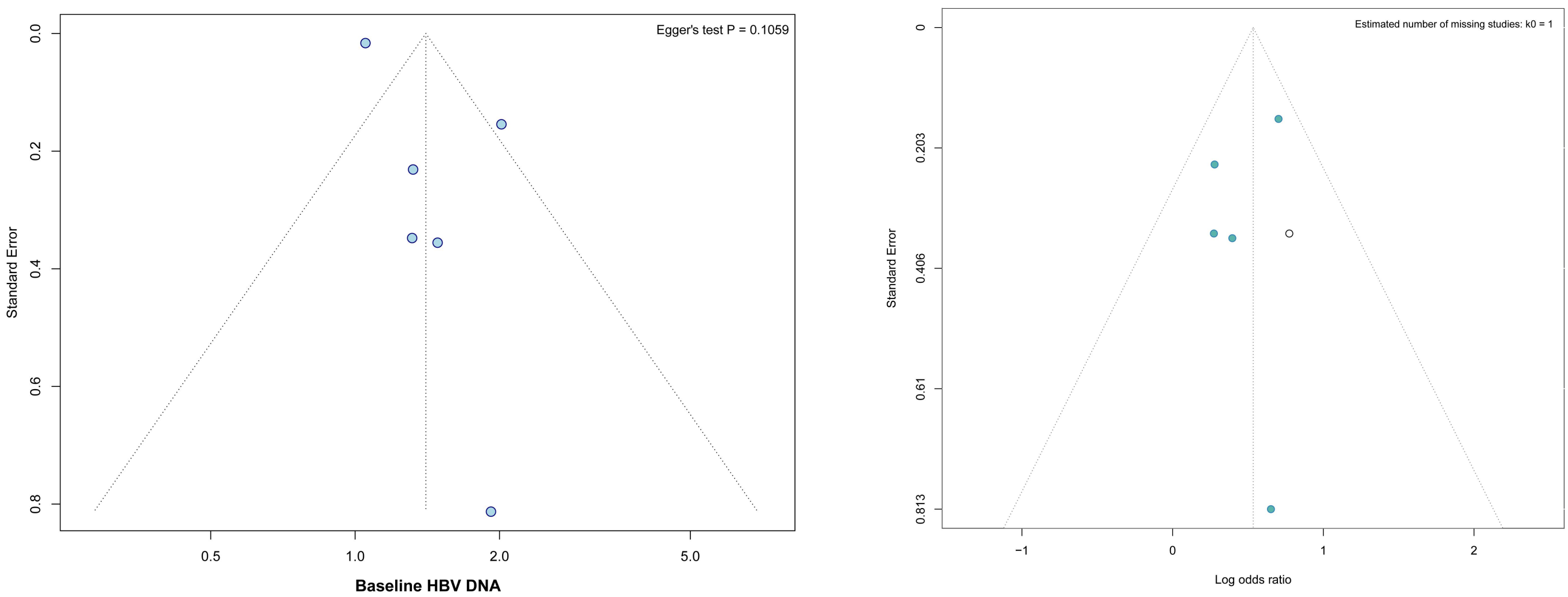

Fig 4. Funnel plot and publication bias assessment using Egger’s regression test and the trim-and-fill method for the association between Baseline HBV DNA and postpartum ALT elevation.

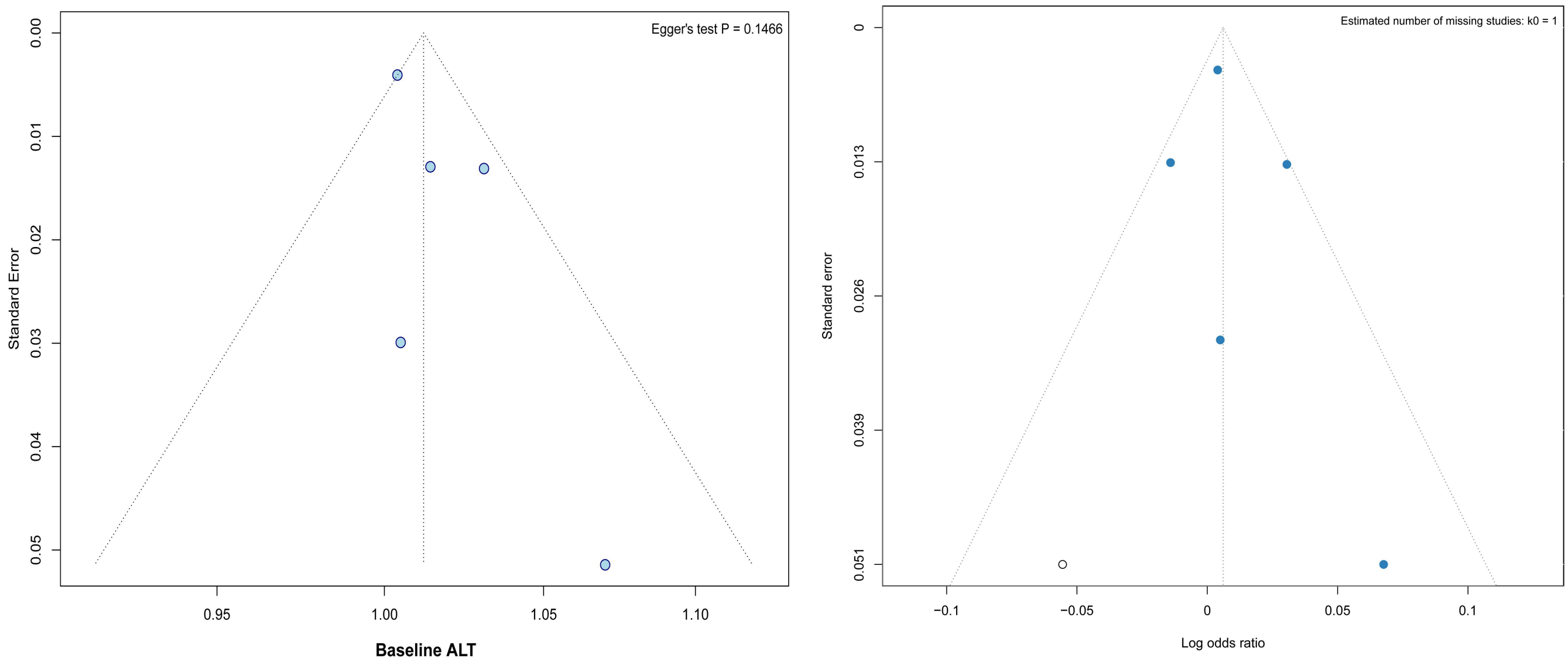

Fig 5. Funnel plot and publication bias assessment using Egger's regression test and the trim-and-fill method for the association between Baseline ALT and postpartum ALT elevation.

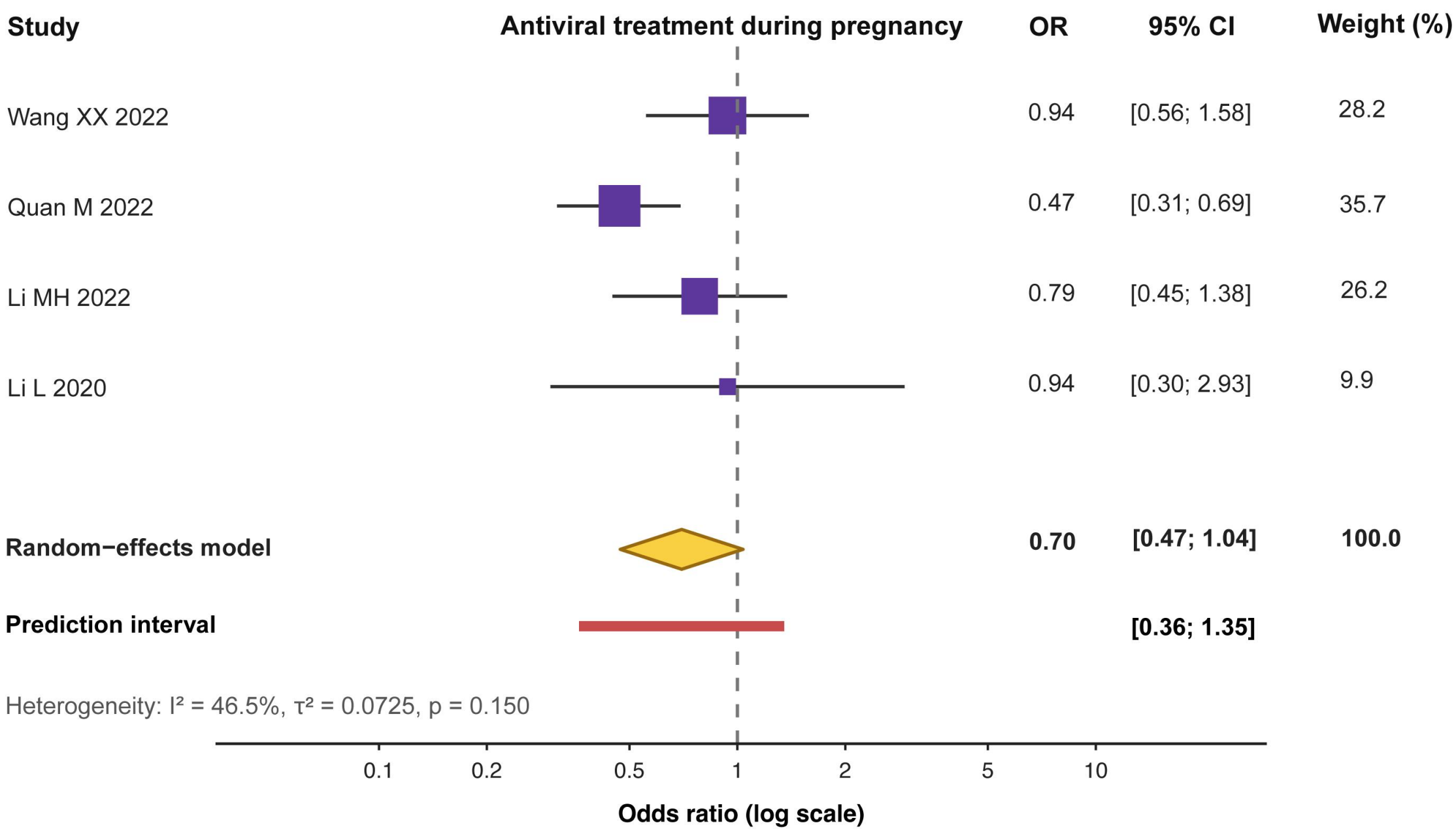

Fig 6. Forest plot and funnel plot of Antiviral treament during pregnancy for postpartum ALT elevation.

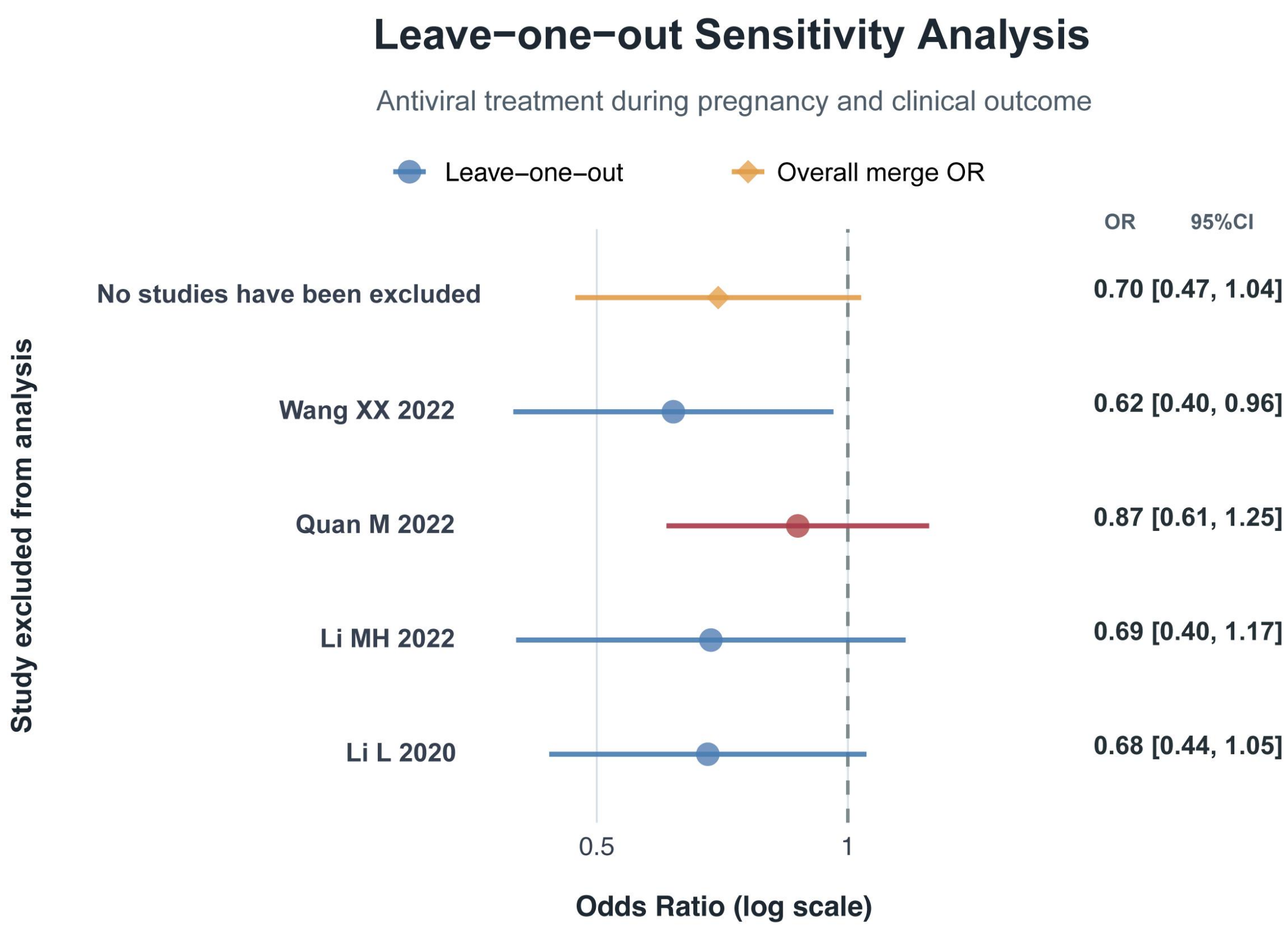

Fig 7. Leave-one-out sensitivity analysis of the meta-analysis of Antiviral treatment during pregnancy.
